# ^7^Be Recovery from Seawater by Sorbents of Various Types

**DOI:** 10.3390/ma16114088

**Published:** 2023-05-31

**Authors:** Nikolay A. Bezhin, Iuliia G. Shibetskaia, Ol’ga N. Kozlovskaia, Evgeniy V. Slizchenko, Ivan G. Tananaev

**Affiliations:** 1 Department of Biogeochemistry, Marine Hydrophysical Institute, Russian Academy of Sciences (MHI RAS), Kapitanskaya Str., 2, 299011 Sevastopol, Russia; nickbezhin@yandex.ru (N.A.B.); iuliia.shibetskaia@gmail.com (I.G.S.); o.n.kozlovska@gmail.com (O.N.K.); evgeniy774@gmail.com (E.V.S.); 2 Department of Chemistry and Chemical Engineering, Sevastopol State University, Universitetskaya Str., 33, 299053 Sevastopol, Russia; 3 Radiochemistry Laboratory, Vernadsky Institute of Geochemistry and Analytical Chemistry of the Russian Academy of Sciences (GEOKHI RAS), Kosygin Str., 19, 119991 Moscow, Russia; 4 Department of Nuclear Technology, Far Eastern Federal University, Sukhanov Str., 8, 690091 Vladivostok, Russia

**Keywords:** beryllium, ^7^Be, seawater, sorbents, manganese dioxide, iron(III) hydroxide

## Abstract

For the first time, a comprehensive study of sorbents based on manganese dioxide was carried out for beryllium sorption from seawater in laboratory and expeditionary conditions. The possibility of using several commercially available sorbents based on manganese dioxide (Modix, MDM, DMM, PAN-MnO_2_) and phosphorus(V) oxide (PD) for ^7^Be recovery from seawater for solving oceanological problems was evaluated. Beryllium sorption under static and dynamic conditions was studied. The distribution coefficients and dynamic and total dynamic exchange capacities were determined. Sorbents Modix (*K_d_* = (2.2 ± 0.1) × 10^3^ mL/g) and MDM (*K_d_* = (2.4 ± 0.2) × 10^3^ mL/g) showed high efficiency. The dependences of the degree of recovery on time (kinetics) and the capacity of the sorbent on the beryllium equilibrium concentration in solution (isotherm) were established. The data obtained were processed using kinetic models (intraparticle diffusion, pseudo-first and pseudo-second orders, Elovich model) and sorption isotherm equations (Langmuir, Freindlich, Dubinin–Radushkevich). The paper contains results of expeditionary studies to evaluate the sorption efficiency of ^7^Be from large volumes of the Black Sea water by various sorbents. We also compared the sorption efficiency of ^7^Be for the considered sorbents with aluminum oxide and previously obtained sorbents based on iron(III) hydroxide.

## 1. Introduction

The cosmogenic isotope ^7^Be forms in the Earth’s atmosphere under the action of fast protons; the main reaction of formation is ^14^N(p,2α)^7^Be. It is a constituent of BeO and Be(OH)_2_ compounds. These molecules diffuse through the atmosphere until they are sorbed by aerosol particles or captured by raindrops [1,2]. After that, ^7^Be enters the underlying surface, including the world ocean, mainly with atmospheric precipitation.

In oceanological studies, ^7^Be is widely used to study the processes of vertical transport in the ocean’s surface layer [3]. The results of several works [4,5] showed that the vertical distribution of ^7^Be generally reflects the temperature profile: the concentration of ^7^Be in the mixed layer is constant, while in the layers below the thermocline, it decreases exponentially. However, searching for materials that allow the most efficient concentration of ^7^Be from seawater is essential because the ^7^Be activity in seawater is only 1–12 Bq/m^3^, according to works [6,7,8].

Aluminum oxide was the first material proposed by W.B. Silker [9] for ^7^Be recovery from seawater. At high seawater flow rates (about 10 C.V./min (column volumes per minute)), the sorption efficiency of ^7^Be is less than 60%; however, reliable determination of ^7^Be activity on the second adsorber and the sorption efficiency becomes possible. This method was successfully used in works [5,8], and according to the data obtained, a mathematical model of ^7^Be distribution in the surface layer of the Black Sea was built [10].

To recover ^7^Be from seawater, most researchers widely use sorbents based on Fe(OH)_3_ and acrylate fiber [3] or polypropylene cartridges [7,11], which have shown a high sorption efficiency of ^7^Be. In particular, for the filtration and concentration system used in [7] based on cartridges impregnated with Fe(OH)_3_, when passing 4400–5500 L of seawater at a rate of 18.3–29.4 L/min, the radiochemical yield during beryllium sorption was 84.2–91.0%. However, this system was not widely used due to the need to keep the research vessel at the station for taking one sample for 3 to 5 h, while to get a vertical profile, the required sampling time increased by multiplying the number of points in the profile. In addition, polypropylene cartridges have a high hydrodynamic resistance, which can lead to the failure of pumping equipment.

Previously, we obtained several sorbents based on PAN (polyacrylonitrile) fiber and iron(III) hydroxide for the sorption of natural and cosmogenic radionuclides from seawater [10,11]. Their comprehensive study was carried out, the distribution coefficients of beryllium, the dynamic (DEC) and total dynamic exchange capacities (TDEC) of sorbents for beryllium were determined, output sorption curves were constructed [12], and the sorption kinetics and isotherm were studied [13]. The obtained sorbents were tested for radionuclides sorption from seawater [12]. In addition, the parameters of beryllium sorption by obtained sorbents based on PAN fiber and iron(III) hydroxide were compared with aluminum oxide [12,13]—the first material proposed for ^7^Be recovery [9].

However, some works [14,15,16] mention the possibility of recovering ^7^Be from seawater using sorbents based on manganese dioxide. Thus, it was proposed to recover beryllium with several sorbents based on manganese dioxide from artificial seawater [14,15]. Under static conditions at V/m = 1000 mL/g, high distribution coefficients of ^7^Be (K_d_ > 10^3^ mL/g) were obtained on MnO_2_ impregnated on an anion exchange resin Biorad AG 1-X4 Cl form, biogenic MnO_2_, and nanostructured mesoporous MnO_2_.

^7^Be sorption from seawater was evaluated on model particles simulating various types of particulate matter, including MnO_2_. High values of ^7^Be sorption were obtained—97 ± 3% [16].

Thus, it is clearly seen that, at present, a small number of materials have been studied for the sorption of ^7^Be from seawater. Most researchers use either aluminum oxide [7] or sorbents based on iron(III) hydroxide [1].

Therefore, to find the optimal sorbent for recovering ^7^Be from seawater, it was necessary to evaluate the possibility of using commercially available sorbents based on manganese dioxide. Previously, we studied sorbents of this type in detail and characterized them concerning strontium [17,18] and radium isotopes [19] when recovered from seawater.

This work aims to conduct a comprehensive study of the sorption and kinetic characteristics of sorbents based on manganese dioxide and phosphorus(V) oxide for beryllium sorption from seawater. In addition, during our research work, we also needed to compare the sorption efficiency of these sorbents with the previously studied sorbents based on iron(III) hydroxide and aluminum oxide in ^7^Be sorption from large volumes of seawater under expeditionary conditions.

## 2. Materials and Methods

### 2.1. Materials

Table 1 shows detailed characteristics of the used sorbents based on manganese dioxide and phosphorus(V) oxide [17].

Beryllium sulfate (analytically pure grade) produced by AO ReaKhim (Moscow, Russia) was used as an additive.

Aluminon (analytically pure grade), ammonium acetate (analytically pure grade), disodium salt of ethylenediaminetetraacetic acid (analytically pure grade) produced by JSC ReaKhim (Moscow, Russia), and gum arabic produced by Alland & Robert S.A. (Port-Mort, France) were used to determine the concentration of stable beryllium.

For the preparation of calibration solutions, a standard sample of beryllium solution ISS (interstate standard sample) 0352:2002 (LLC Ormet, Ekaterinburg, Russia) was used.

### 2.2. Determination of Beryllium Distribution Coefficients

The distribution coefficients of beryllium were determined according to the unified method proposed in [20]. For this purpose, 20 mL of the test solution was added to 0.1 g of the sorbent. Sea water with the addition of beryllium to a concentration of 0.3 mg/L was used as the test solution. The resulting mixture was kept for 48 h with occasional stirring, then separated by filtration. Each experiment was repeated at least three times.

### 2.3. Sorption Dynamics

To study the dynamics of sorption through a column filled with 3 g of the sorbent, seawater with the addition of beryllium 0.3 mg/L was passed at different rates (3, 6, 15, 30 mL/min) using a LongerPump WT600-2J peristaltic pump (Longer Precision Pump Co., Baoding, China). After certain time intervals, a portion of the eluate was taken to estimate the number of beryllium ions that had not been sorbed. Each experiment was repeated at least three times for each rate.

### 2.4. Sorption Kinetics

The dependence of the degree of beryllium recovery on the time of sorption was determined by mixing 0.1 g of the sorbent with 10 mL of seawater with the addition of beryllium 0.3 mg/L for various times from 1 to 48 h. Then, the mixture was separated by filtration. Each experiment was repeated at least three times for each time point.

### 2.5. Sorption Isotherm

Determination of the dependence of the capacity of sorbents on the equilibrium concentration of beryllium in solutions was carried out by mixing 0.1 g of the sorbent with 10 mL of seawater with various additions of beryllium for 48 h. Then, the mixture was separated by filtration. Each experiment was repeated at least three times for each concentration.

### 2.6. Determination of the Concentration of Beryllium in Solution and Quantitative Parameters of Sorption

The beryllium concentration in solutions before and after sorption was determined using a KFK-3-01 photometer (JSC Zagorsk Optical and Mechanical Plant, Sergiev Posad, Russia) according to the method [21]. For this purpose, an aluminon solution was prepared by dissolving 0.5 g of aluminon, 140 g of ammonium acetate, and 10 g of gum arabic in distilled water, after which the volume of the solution was brought up to 1 L with distilled water. The resulting solution was filtered. A total of 5 mL of the disodium salt of ethylenediaminetetraacetic acid solution (5 g in 100 mL of water) and 10 mL of aluminon solution were added to 25 mL of the analyzed solution in a 50 mL volumetric flask. The mixture was heated for 10 min in a water bath, quickly cooled to room temperature (under cold water flow), and diluted with distilled water to the mark. Standard solutions with beryllium concentrations of 0.1, 0.2, and 0.4 mg/L were prepared using ISS Be 0352:2002 and with the same reagents as the analyzed samples. Optical density was measured at 536 nm in a 2 cm cuvette using a blank sample prepared with reagents, as well as the analyzed samples. The relative error in the determination of beryllium was 2–4%.

The distribution coefficient (*K_d_*, mL/g), the degree of recovery (*R*, %), the sorbents capacity (*q*, mg/g), and the dynamic (DEC, mg/g) and total dynamic exchange capacity (TDEC, mg/g) were determined by the equations given in the articles [12,17].

### 2.7. Sorption of ^7^Be from Large Volumes of Seawater

Seawater sampling was carried out in the Black Sea during the 116 cruises (22 April–17 May 2021) of the R/V Professor Vodyanitsky using a UNIPUMP BAVLENETS BV 0.12-40-U5 submersible vibration pump (LLC Subline Service, Moscow, Russia) into plastic tanks located on board. In the process of sampling, for separation of suspended matter, the sample was filtered through 5 inches high polypropylene cartridges with a pore size of 0.5 µm FCPS1M series (Aquafilter Europe Ltd., Lodz, Poland) at a rate of 6–10 L/min. A high filtration rate is necessary since the prefilter saturated with suspended matter acts as a “sorbent” for several radionuclides, in particular ^7^Be, which misrepresents the results of its initial activity in dissolved and suspended form.

Sorption of ^7^Be from large volumes of seawater was performed using single-column and two-column methods.

The one-column method consisted in adding stable beryllium as a tracer to the seawater sample to a concentration of 0.3 mg/L. 5–6 h later, when the concentration of beryllium was equalized in the entire volume of the container, the sample was passed through one column with the studied sorbent. In this case, every 10–50 L, a sample of the passed seawater was taken to analyze the concentration of stable beryllium in it for further evaluation of the sorption efficiency using the equation given in [22,23]:

(1) $$E=\frac{V\cdot C_{0}-\sum V_{p}\cdot C_{p}}{V\cdot C_{0}}\cdot 100\%$$

where *C*_0_ is the initial cesium concentration, mg/L; *V* is the total volume of seawater passed through the sorbent, L; *C_p_* is the cesium concentration in a portion of seawater passed through the sorbent, mg/L; and *V_p_* is the volume of a portion of seawater passed through the sorbent, L.

The two-column method consisted of passing seawater samples through two successively installed columns with studied sorbent of the same volume. The sorption efficiency was determined by the equation given in [24]:

(2) $$E=1-\frac{B}{A}$$

where *A* and *B* are the activities of the radionuclide on the sorbent in the first and second adsorbers.

The disadvantage of the method is a significant error due to the low activity of the extracted radionuclide on the second sorbent.

The sample volume for extracting ^7^Be ranges from 250 to 1000 L and depends on the time interval between the concentration and sample measurement since the half-life of ^7^Be is only 53.3 days. In a long expedition, the time interval between concentration and measurement of the samples can be more than a month, and then larger samples are needed.

After ^7^Be concentration, the sorbents were dried in a SNOL 58/350 I4 oven (AB UMEGA-GROUP, Utena, Lithuania) at a temperature of 70–80 °C. When using fibrous sorbents, after drying, the sorbent was additionally ashed in a SNOL-30/1300-I1p muffle furnace (AB UMEGA-GROUP, Utena, Lithuania) at 700 °C for 8 h to reduce the volume of the counting sample and as a consequence, self-absorption of gamma radiation.

### 2.8. Determination of ^7^Be Activity in Sorbents

^7^Be activity in the sorbents was determined by the 477 keV (10.44%) photopeak [25] on a Canberra multichannel gamma spectrometer for measuring X-ray and gamma radiation (Canberra Industries, Meriden, CT, USA) with Canberra BE3825 Broad Energy High Purity Germanium Detector for at least 24 h. In this case, the relative error measurement was 13–16%. Detection efficiency was estimated using ISOCS/LabSOCS software (Canberra Industries, Meriden, CT, USA).

## 3. Results and Discussion

### 3.1. Beryllium Distribution Coefficients

Table 2 shows the determined distribution coefficients of beryllium from seawater by the studied sorbents.

It is clear that beryllium is more effectively sorbed on MDM and Modix sorbents. In general, the sorbents under study can be arranged in the following order in terms of the beryllium distribution coefficient:

MDM ≈ Modix > PAN-MnO_2_ > PD ≈ DMM.

For beryllium, the distribution coefficients obtained on sorbents on manganese dioxide are generally higher than the distribution coefficients obtained in our study [12] on sorbents based on iron(III) hydroxide. For example, among the sorbents of this type, the best distribution coefficient of beryllium was obtained on the Fe-SF sorbent (Fe-Sodium Ferrate, obtained using ready-made Na_2_FeO_4_) and amounted to (1.1 ± 0.1)×10^3^ mL/g (Table S1).

These results are due to the fact that Modix and MDM sorbents are granular manganese dioxide, differing only in particle size: 0.16–0.3 mm and 0.2–3.0 mm, respectively. At the same time, the other sorbents under consideration, including those based on iron hydroxide (III), consist of a carrier on which an active component is applied; its content in the finished sorbent varies from 5 to 22% (Table 1).

### 3.2. Sorption Dynamics

**Figure 1:**
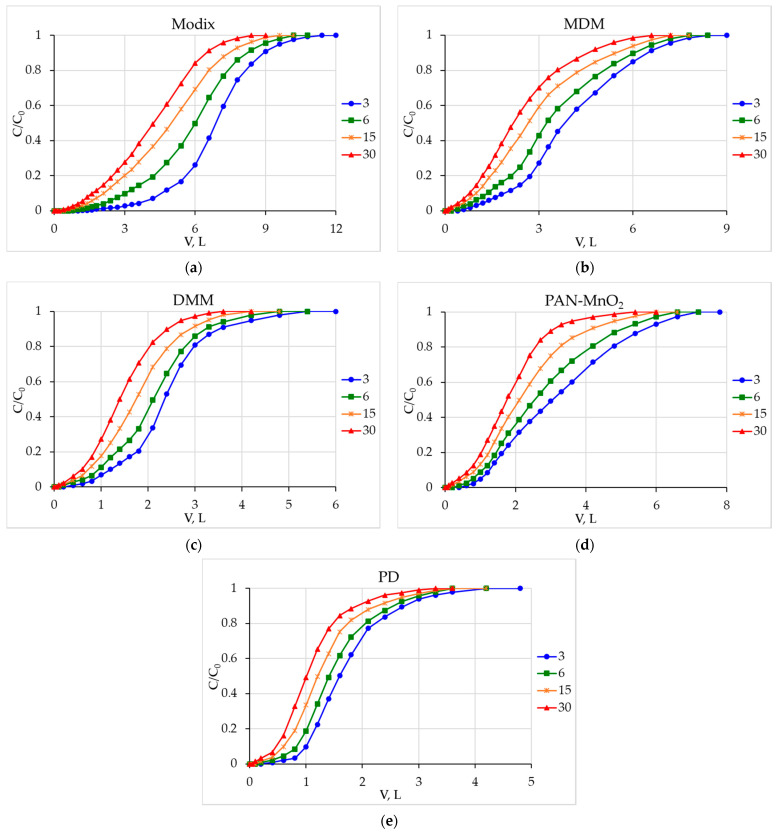
Beryllium sorption output curves at different flow rates (3, 6, 15, and 30 mL/min) on the sorbents: (**a**) Modix; (**b**) MDM; (**c**) DMM; (**d**) PAN-MnO_2_; (**e**) PD.

Figure 1 shows beryllium recovery output curves obtained at various seawater flow rates.

The shapes of the output curves clearly show that the Modix sorbent has higher capacity characteristics. A more S-shaped output curve is characteristic of sorbents with higher values of exchange capacity; a more convex output curve indicates low values of exchange capacity.

Table 3 shows the obtained values of DEC and TDEC.

The DEC and TDEC of the Modix sorbent were higher than these of the other sorbents. In general, the studied sorbents can be arranged in the following order in terms of DEC and TDEC:

Modix > MDM > PAN-MnO_2_ > DMM > PD.

The DEC and TDEC values of the previously studied sorbents based on iron(III) hydroxide [12] were generally lower than the values obtained for the Modix sorbent. In particular, the best Fe-H sorbent (Fe-Hydrolyzed, obtained using pre-hydrolyzed PAN with precipitation of Fe(OH)_3_ by ammonia) in terms of these characteristics at a seawater flow rate of 3 mL/min had somewhat lower DEC and TDEC values of 0.068 and 0.656 mg/g (Table S1), respectively [12].

### 3.3. Sorption Kinetics

**Figure 2:**
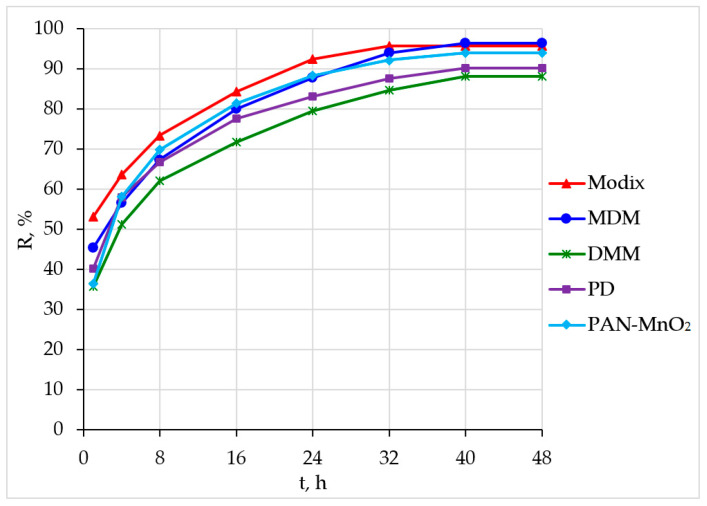
Kinetic curves of beryllium sorption.

Figure 2 shows the results of determining the dependence of the degree of recovery of beryllium on the time of sorption.

It is clear that the studied sorbents reach the time sorption equilibrium within 40–48 h, which corresponds to the data on the sorption kinetics on aluminum oxide and sorbents on iron(III) hydroxide, except the Fe-H sorbent, which extracts beryllium in 10 min [13].

The obtained data were processed using four kinetic models: pseudo-first [26,27,28,29] and pseudo-second [26,27,28,30] orders, intraparticle diffusion [26,30], and the Elovich model [27,28,31].

The obtained experimental values of the equilibrium capacity of the sorbents correspond to the values obtained by the pseudo-second-order equation (Table 4).

The acquired parameters of the kinetic models generally correspond to the parameters obtained on sorbents based on iron(III) hydroxide [13] and are well described using the pseudo-second-order model, which makes it possible to take into account not only sorbate–sorbent interactions, but also intermolecular interactions of adsorbed substances. Thus, the chemical exchange reaction limits the sorption process [26,27,30].

In general, changes in the sorption rate constants of the models pseudo-first (*K*_1_) and pseudo-second-order (*K*_2_) and Elovich model (α) have common patterns for sorbents based on MnO_2_:

Modix > PAN-MnO_2_ > MDM > DMM.

### 3.4. Sorption Isotherm

**Figure 3:**
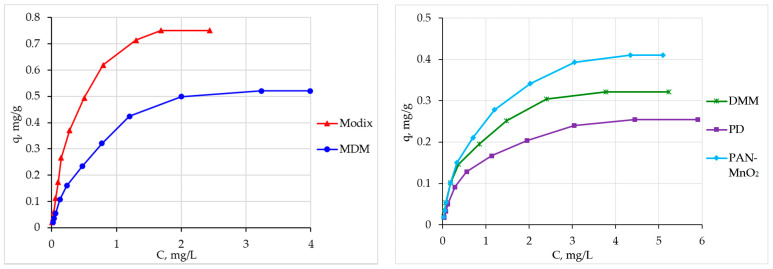
Sorption isotherms of beryllium.

Figure 3 shows the determined dependences of the capacity of sorbents on the equilibrium concentration of beryllium in solutions. It is seen that the maximum beryllium capacity of sorbents is reached at an equilibrium beryllium concentration in a solution of more than 2 mg/L. The Modix sorbent (0.752 mg/g) has the highest beryllium capacity, which exceeds the maximum capacity previously obtained for the Fe-H sorbent (0.694 mg/g, Table S1) [13].

The obtained data were processed using the Langmuir [26,29,31], Freindlich [27,29,31], and Dubinin–Radushkevich [26,31,32,33] sorption equations.

The obtained experimental values of the maximum capacity of the sorbents correspond to the values obtained from the Langmuir isotherm equation (Table 5), which describes adsorption on a monomolecular layer well. Thus, the obtained data on the recovery of beryllium by all the studied sorbents are well described by the Langmuir sorption isotherm equation, which corresponds to the results obtained on sorbents based on iron(III) hydroxide [13].

### 3.5. Sorption of ^7^Be from Large Volumes of Seawater

**Figure 4:**
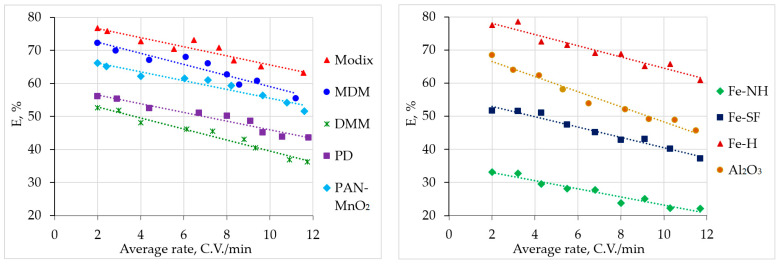
Sorption efficiency of ^7^Be from large volumes of seawater by sorbents of various types.

Figure 4 shows the results of evaluating the sorption efficiency of ^7^Be from seawater samples of large volume by the studied sorbents in comparison with the sorbents based on iron(III) hydroxide and aluminum oxide presented in [12]. In our work [12], a detailed study of the sorption efficiency of ^7^Be by sorbents based on iron(III) hydroxide and aluminum oxide was not carried out; only the results of single tests were presented when 1000 L of seawater were passed through 2 columns of 100 g of the sorbent at a rate of 1 L/min.

According to the obtained results, the best efficiency of ^7^Be sorption from seawater is shown by Modix (*E* = 63.2–76.7%), MDM (*E* = 55.5–72.3%), and Fe-H (*E* = 60.9–78.5%) sorbents. The optimal seawater flow rate for ^7^Be sorption is 2–6 C.V./min. Examples of the obtained ^7^Be activities in the Black Sea water in the spring of 2021 when testing various sorbents at a seawater transmission rate of 2.4–3.2 C.V./min are given in Table S2.

Thus, these sorbents can be successfully used to solve the oceanological problem of estimating vertical transport [34,35].

In the course of laboratory tests, sorbents based on manganese dioxide proved to be more efficient in the recovery of beryllium compared to sorbents based on iron(III) hydroxide according to [12,13]; however, in the expeditionary studies, their efficiency is comparable to sorbents based on iron(III) hydroxide.

This is explained by the fact that sorbents based on manganese dioxide, in contrast to sorbents based on iron(III) hydroxide and also sorb radium isotopes from seawater quite efficiently [36,37,38,39,40].

Thus, sorbents based on manganese dioxide can be effectively used to recover such isotopes important for oceanological research [41] as ^7^Be, ^210^Pb [14,15,39,40], ^210^Po [14,15,40], “radium quartet” (^223^Ra, ^224^Ra, ^226^Ra, ^228^Ra) [36,37,38,39,40], ^234^Th [14,15,39] from seawater. The gamma spectrometric method makes it possible to determine the activity of most of these isotopes (^7^Be [25], ^210^Pb [42], ^226^Ra and ^228^Ra [43], ^234^Th [44]) from various peaks. Determination of ^210^Po is possible by the alpha spectrometric method [45] after radiochemical preparation; short-lived radium isotopes (^223^Ra, ^224^Ra) can be detected using the RaDeCC (Radium Delayed Coincidence Counter) system [46]. However, ^226^Ra can only be measured 3 weeks after hermetic sealing to achieve its equilibrium with ^214^Pb [43].

Moreover, it is possible effectively and reliably separate the peaks of beryllium and radium due to their superposition (Figures S1 and S2) only on gamma spectrometric equipment with HPGe detectors, which can be difficult for several laboratories with less efficient equipment.

Sorbents based on iron(III) hydroxide can be effectively used to recover such isotopes as ^7^Be [3,7,11,12,47], ^32^P, and ^33^P [7,11,12,47], ^210^Pb [12,48,49,50], ^210^Po [51], ^234^Th [12,47,50] from seawater. The isotopes ^7^Be [25], ^210^Pb [42], and ^234^Th [44] can be simultaneously analyzed on a gamma spectrometer using different peaks, and there are no problems in separating the peaks of thorium and radium, which makes it possible to use even scintillation gamma rays for effective analysis of ^7^Be and ^234^Th spectrometers. Analysis of ^32^P, ^33^P, and ^210^Po requires additional radiochemical preparation. Unfortunately, for a pair of phosphorus and ^210^Po isotopes, the radiochemical preparation is different, so their simultaneous analysis is not possible. After radiochemical preparation, ^32^P and ^33^P can be analyzed by beta spectrometry [52] and ^210^Po—by alpha spectrometry [45].

We assume that in the case of gamma-spectrometric determination of the activity of radionuclides in sorbent samples, it is most effective to carry out a complex extraction of radionuclides by sequentially arranging the first columns of sorbents based on iron(III) hydroxide and then columns of sorbents based on manganese dioxide. Thus, it will be possible to determine ^7^Be, ^210^Pb, and ^234^Th on a sorbent based on iron(III) hydroxide and ^226^Ra and ^228^Ra on the next sorbent based on manganese dioxide. We use this principle in the sequential arrangement of PAN-Fe(OH)_3_ and PAN-MnO_2_ sorbents in our new studies.

Despite high sorption efficiency, the Modix sorbent has a high hydrodynamic resistance due to the small size of the granules (0.16–0.3 mm). However, in expeditionary studies, to reduce the analysis time and increase the number of samples taken, it is important to achieve a high rate of seawater flowing through the sorbent. Therefore, at high seawater flow rates, sorbents with relatively large granules would be the best choice, even though they are less efficient, for example, MDM (granule size 1.0–3.0 mm). Fibrous sorbents modified with manganese dioxide or iron(III) hydroxide have low hydrodynamic resistance and proved good for the recovery of radionuclides in expeditionary studies.

## 4. Conclusions

Thus, sorbents based on manganese dioxide and phosphorus(V) oxide to extract ^7^Be from seawater can be used for solving the problems of oceanology. A comprehensive study of commercially available sorbents, from laboratory static and dynamic tests to expeditionary studies on large volumes of seawater, was carried out. The results obtained on sorbents based on manganese dioxide were compared with sorbents based on iron(III) hydroxide.

Experimental data on the distribution coefficients of beryllium on the studied sorbents and the values of dynamic and total dynamic exchange capacities were obtained. The obtained values for beryllium exceeded those for sorbents based on iron(III) hydroxide.

Isotherms, output, and kinetic curves of sorption were plotted. The obtained data were characterized using several sorption models. A pseudo-second-order kinetic model (r^2^ > 0.996) described well the determined dependences of the degree of recovery on the sorption time; the Langmuir sorption isotherm (r^2^ > 0.997) well described the dependence of the sorbent capacity on the equilibrium concentration of beryllium.

In tests on large volumes of seawater, the efficiency of sorbents based on manganese dioxide (*E* = 63.2–76.7% for Modix, *E* = 55.5–72.3% for MDM) was comparable to sorbents based on iron(III) hydroxide (*E* = 60.9–78.5% for Fe-H), which is explained by more efficient specific sorption of radium isotopes from seawater by sorbents based on manganese dioxide.

## Figures and Tables

**Table 1:** Characteristics of the sorbents used for beryllium separation.

Sorbent	Manufacturer	Appearance	Grain Size, mm	Bulk Weight, g/mL	Sorbent Composition	
					Support	Sorption-Active Phase: Content, Mass %
Modix	SPE Eksorb Ltd. (Ekaterinburg, Russia)	Irregular-shaped black granules	0.16–0.3	0.6–0.9	–	manganese dioxide
MDM	SPE Ektos-Atom, JSC (Moscow, Russia)	Irregular-shaped dark brown granules	0.1–3.0	0.4–0.6	–	manganese oxides (III, IV)
DMM		Irregular-shaped dark brown granules	0.1–5.0	0.1–0.4	cellulose	manganese oxides (III, IV); not less than 5
PD		Irregular-shaped light brown granules	0.1–5.0	0.1–0.4	phosphorylated wood	phosphorus(V) oxide; not less than 13
PAN-MnO_2_	–	black fiber	–	–	PAN fiber	manganese dioxide; 18–22

**Table 2:** Beryllium distribution coefficients.

Sorbent	Modix	MDM	DMM	PAN-MnO_2_	PD
*K_d_*, mL/g	(2.2 ± 0.1) × 10^3^	(2.4 ± 0.2) × 10^3^	700 ± 50	(1.5 ± 0.1) × 10^3^	850 ± 90

**Table 3:** DEC and TDEC of sorbents for beryllium.

Sorbent	Flow Rate, mL/min							
	3		6		15		30	
	DEC, mg/g	TDEC, mg/g	DEC, mg/g	TDEC, mg/g	DEC, mg/g	TDEC, mg/g	DEC, mg/g	TDEC, mg/g
Modix	0.125	0.739	0.093	0.637	0.062	0.541	0.042	0.470
MDM	0.062	0.460	0.042	0.407	0.021	0.346	0.010	0.298
DMM	0.042	0.295	0.031	0.267	0.016	0.234	0.006	0.171
PAN-MnO_2_	0.052	0.378	0.035	0.334	0.018	0.284	0.008	0.266
PD	0.031	0.229	0.021	0.180	0.012	0.157	0.004	0.135

**Table 4:** Parameters of kinetic models.

Sorbent	Intraparticle Diffusion *			Pseudo-First- Order *			Pseudo-Second-Order *			Elovich Model *			*q_e exp_*, mg/g
	*K_I_*, mg/(g∙h^0.5^)	*c*, mg/g	*r* ^2^	*K*_1_, h^−1^	*g*_e_, mg/g	*r* ^2^	*K*_2_, g/(mg∙h)	*g*_e_, mg/g	*r* ^2^	*α*, g/(mg∙h)	*β*, g/mg	*r* ^2^	
Modix	0.0019	0.0129	0.933	0.108	0.0132	0.980	18.3	0.0259	0.998	0.136	313	0.974	0.0247
MDM	0.0023	0.0103	0.965	0.093	0.0158	0.973	11.8	0.0264	0.996	0.065	270	0.975	0.0249
DMM	0.0023	0.0086	0.954	0.083	0.0142	0.988	12.0	0.0242	0.996	0.039	270	0.992	0.0228
PAN-MnO_2_	0.0024	0.0098	0.904	0.104	0.0156	0.991	13.8	0.0257	0.999	0.044	250	0.994	0.0243
PD	0.0021	0.0103	0.930	0.090	0.0132	0.990	15.3	0.0245	0.998	0.066	286	0.997	0.0233

* *K_I_* is the rate constant of intraparticle diffusion, mg/(g∙h^0.5^); *c* is the constant characterizing the contribution of the boundary layer, mg/g; *q_e_* is the equilibrium capacity of the sorbent, mg/g; *K*_1_ is the pseudo-first-order rate constant, h^−1^; *K*_2_ is the pseudo-second-order rate constant, g/(mg∙h); α is the initial sorption rate constant, g/mg h; *β* is the desorption constant, g/mg; *r*^2^ is the approximation confidence factor.

**Table 5:** Parameters of sorption isotherms.

Sorbent	Langmuir Isotherm *			Freindlich Isotherm *			Dubinin–Radushkevich Isotherm *				*q_m exp_*, mg/g
	*q_m_*, mg/g	*K_L_*, L/mg	*r* ^2^	*K_F_*, mg/g	*n*	*r* ^2^	*q_m_*, mg/g	*β,* mol^2^/kJ^2^	*E,* kJ/mol	*r* ^2^	
Modix	0.761	2.91	0.997	0.646	1.50	0.956	1.01	0.0138	6.02	0.996	0.752
MDM	0.530	1.95	0.999	0.314	1.60	0.963	0.600	0.0152	5.74	0.997	0.521
DMM	0.323	2.58	0.999	0.186	1.94	0.952	0.353	0.0135	6.09	0.998	0.321
PAN-MnO_2_	0.415	1.83	0.999	0.215	1.75	0.963	0.441	0.0152	5.74	0.998	0.410
PD	0.256	2.07	0.999	0.136	1.96	0.954	0.272	0.0146	5.85	0.999	0.254

* *q_m_* is the maximum capacity of the sorbent, mg/g; *K_L_* is the Langmuir adsorption equilibrium constant, L/mg; *K_F_* is the Freundlich constant, mg/g; n is the empirical indicator of the heterogeneity of exchange centers; *β* is the constant associated with sorption energy, mol^2^/kJ^2^; *E* is the average free energy of sorption, kJ/mol; *r*^2^ is the approximation confidence factor.

## Data Availability

Not applicable.

## Supplementary Materials

The following supporting information can be downloaded at: https://www.mdpi.com/article/10.3390/ma16114088/s1, Figure S1. Gamma spectrum of a sorbent based on manganese dioxide, obtained by radionuclides sorption from seawater by a single-column method. Figure S2. Gamma spectra of sorbents based on iron(III) hydroxide, obtained by radionuclides sorption from seawater by a two-column method. Table S1. Parameters of beryllium sorption by sorbents based on iron(III) hydroxide and aluminum oxide. Table S2. Examples of the obtained ^7^Be activities in the Black Sea water in the spring of 2021 when testing various sorbents.
