# Supplementary material for: 7Be Recovery from Seawater by Sorbents of Various Types
Source: Materials (Basel). 2023 May 31;16(11):4088. doi: 10.3390/ma16114088 (PMC10254913; doi:10.3390/ma16114088)
Supplement: Supplementary file 1 [file materials-16-04088-s001.zip › materials-2376295-supplementary.pdf]

materials

MDPI

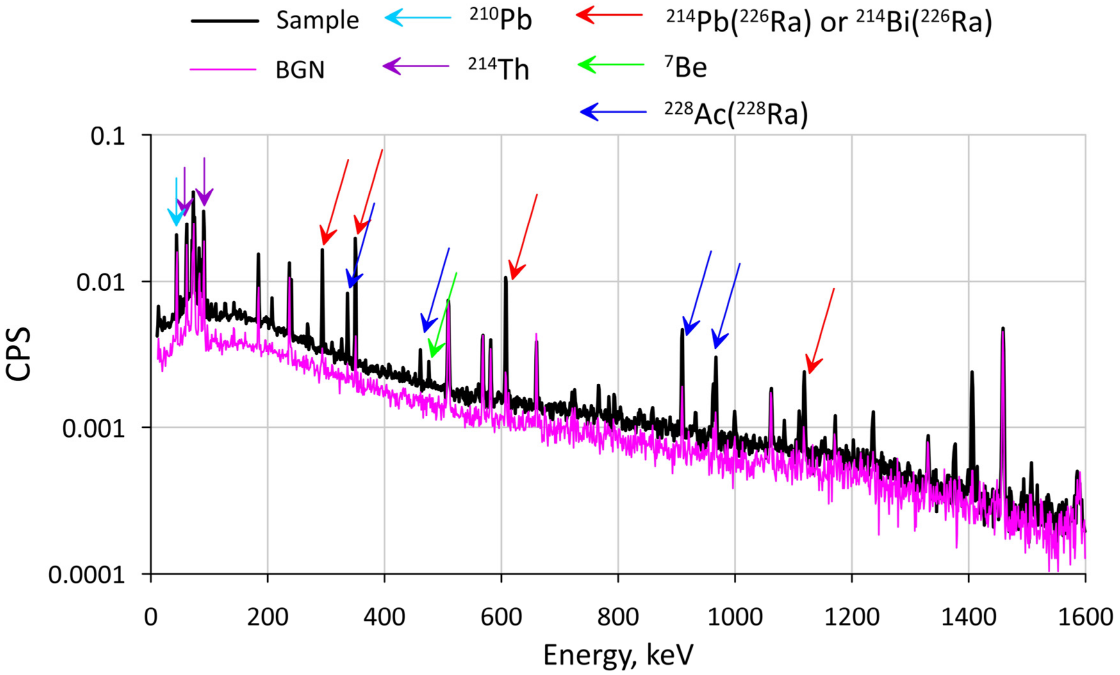

Figure S1. Gamma spectrum of a sorbent based on manganese dioxide, obtained by radionuclides sorption from seawater by a single-column method (BGN – background).

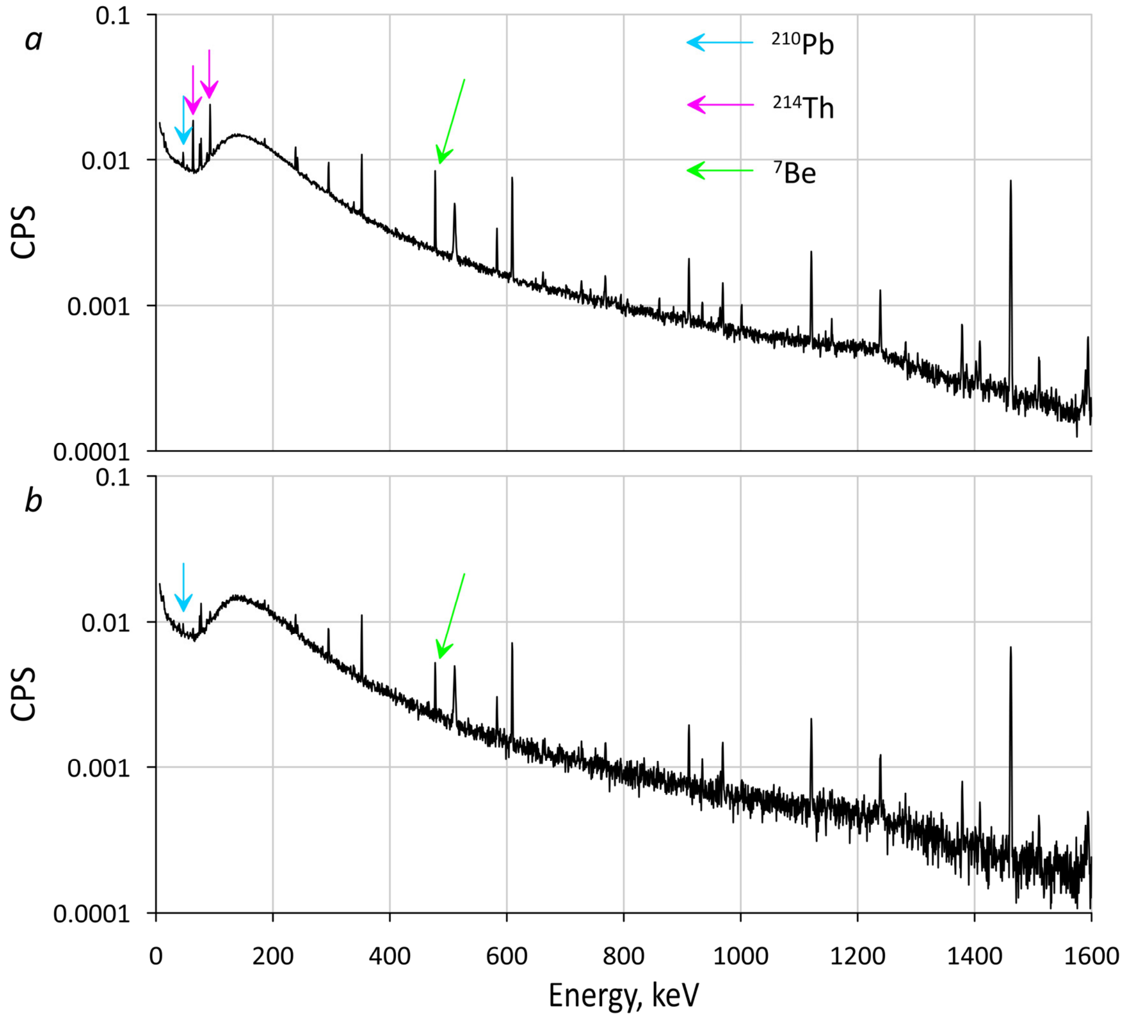

Figure S2. Gamma spectra of sorbents based on iron(III) hydroxide, obtained by radionuclides sorption from seawater by a two-column method (a – the first sorbent (column), b – the second sorbent (column)).

Table S1. Parameters of beryllium sorption by aluminum oxide and sorbents based on iron(III) hydroxide.

Sorbent	Fe-NH	Fe-EGSF	Fe-SF	Fe-H	Al_2O_3
K_d, mL/g	520 ± 70	650 ± 80	(1,1 ± 0,1)×10³	740 ± 85	830 ± 80
DEC, mg/g	0.004	0.035	0.069	0.068	0.004
TDEC, mg/g	0.055	0.240	0.311	0.656	0.044
$q_{m\ exp}$, mg/g	0.096	0.304	0.390	0.694	0.081

Table S2. Examples of the obtained ^{7}Be activities in the Black Sea water in the spring of 2021 when testing various sorbents.

**Sorbent**	**Modix**	**MDM**	**DMM**	**PAN-MnO_2**	**PD**	**Fe-SF**	**Fe-H**	**Al_2O_3**
Transmission rate, C.V./min	2,45	2,85	2,98	2,40	2,89	3,20	3,20	3,00
E, %	75,8	69,9	51,9	65,1	55,3	51,5	78,5	64,0
Activity, Bq/m^3	5,83 ± 0,76	5,47 ± 0,71	5,01 ± 0,75	6,08 ± 0,85	4,72 ± 0,68	6,15 ± 0,92	6,42 ± 0,84	3,94 ± 0,55
